# Strain-resolved microbiome sequencing reveals mobile elements that drive bacterial competition on a clinical timescale

**DOI:** 10.1186/s13073-020-00747-0

**Published:** 2020-05-29

**Authors:** Soumaya Zlitni, Alex Bishara, Eli L. Moss, Ekaterina Tkachenko, Joyce B. Kang, Rebecca N. Culver, Tessa M. Andermann, Ziming Weng, Christina Wood, Christine Handy, Hanlee P. Ji, Serafim Batzoglou, Ami S. Bhatt

**Affiliations:** 1 Departments of Genetics, Stanford University, Stanford, CA USA; 2 Department of Medicine, Division of Hematology, Stanford University, 269 Campus Drive, MC5156, Stanford, CA 94305 USA; 3 Department of Computer Science, Stanford University, Stanford, CA USA; 4 Harvard Medical School, Boston, MA USA; 5 Department of Medicine, Division of Infectious Diseases, University of North Carolina, Chapel Hill, USA; 6 Department of Pathology, Stanford University School of Medicine, Stanford, CA USA; 7 Division of Oncology, Department of Medicine, Stanford University, Stanford, CA USA

**Keywords:** Metagenomics, Read cloud assembly, Strain diversity, Gut microbiome, Sequencing, DNA, Antibiotic resistance, Linked reads, Structural variation

## Abstract

**Background:**

Populations of closely related microbial strains can be simultaneously present in bacterial communities such as the human gut microbiome. We recently developed a de novo genome assembly approach that uses read cloud sequencing to provide more complete microbial genome drafts, enabling precise differentiation and tracking of strain-level dynamics across metagenomic samples. In this case study, we present a proof-of-concept using read cloud sequencing to describe bacterial strain diversity in the gut microbiome of one hematopoietic cell transplantation patient over a 2-month time course and highlight temporal strain variation of gut microbes during therapy. The treatment was accompanied by diet changes and administration of multiple immunosuppressants and antimicrobials.

**Methods:**

We conducted short-read and read cloud metagenomic sequencing of DNA extracted from four longitudinal stool samples collected during the course of treatment of one hematopoietic cell transplantation (HCT) patient. After applying read cloud metagenomic assembly to discover strain-level sequence variants in these complex microbiome samples, we performed metatranscriptomic analysis to investigate differential expression of antibiotic resistance genes. Finally, we validated predictions from the genomic and metatranscriptomic findings through in vitro antibiotic susceptibility testing and whole genome sequencing of isolates derived from the patient stool samples.

**Results:**

During the 56-day longitudinal time course that was studied, the patient’s microbiome was profoundly disrupted and eventually dominated by *Bacteroides caccae*. Comparative analysis of *B. caccae* genomes obtained using read cloud sequencing together with metagenomic RNA sequencing allowed us to identify differences in substrain populations over time. Based on this, we predicted that particular mobile element integrations likely resulted in increased antibiotic resistance, which we further supported using in vitro antibiotic susceptibility testing.

**Conclusions:**

We find read cloud assembly to be useful in identifying key structural genomic strain variants within a metagenomic sample. These strains have fluctuating relative abundance over relatively short time periods in human microbiomes. We also find specific structural genomic variations that are associated with increased antibiotic resistance over the course of clinical treatment.

## Background

Microbial strains of the same species share “core” genes that encode conserved functions common to the species. However, strains of a given species can differ by single-nucleotide variants (SNV), insertions, and deletions as well as structural arrangement [1] and variable presence of accessory genes [2, 3], which facilitate adaptability of the species population as a whole. Strain-level variation can arise from several mechanisms including horizontal gene transfer and transposon mobilization. Each of these mechanisms has a well-described capacity to induce significant changes in phenotype. Through horizontal gene transfer, bacteria can acquire and disseminate genomic elements encoding antibiotic resistance genes, virulence factors, or metabolic capabilities [4, 5]. Other mobile elements, such as transposons, can affect gene function and regulation by either disrupting coding sequences [6], or by upregulating neighboring genes through the introduction of strong promoter sequences often carried with the transposon [7, 8]. These transposons can be mobilized during physiological stress, such as exposure to antibiotics, and this mobilization can result in acquisition of improved niche-specific fitness [9].

Genetic variation within a population can either arise de novo as a consequence of new mutation or can be part of a reservoir of genetic variation in a community where multiple strains stably coexist [10]. Exposure to various selective pressures from the environment can trigger the rise of de novo mutations within members of a population. Eventually, alleles that confer a selective advantage are fixed and strains carrying these alleles persist. Alternatively, genetic heterogeneity within a microbial species can be advantageous with a change in the environment. Positive selection takes place when a strain variant carrying a beneficial allele is favored by natural selection given a change in the environment (e.g., exposure to antibiotics). With that, the frequency of the fit variant increases over time until it eventually sweeps the population. While significant advances have been made in defining species- and genus-level taxonomic composition of the human microbiome, much remains to be understood about sub-species or strain-level genetic diversity, how it arises, and more importantly whether strain heterogeneity has compositional and functional implications on the community. Recent advances in microbial sequencing and computational tools for genome assembly and annotation are opening new avenues to study the genetic diversity of microbial species and understand the functional consequences of such diversity.

Shotgun short-read sequencing methods facilitate study of the genomic content and strain-level architecture of complex microbial communities. Comparisons of microbial genomes obtained from both sequencing of isolates and single cells have shaped our understanding of strain-level genetic variability [5, 11, 12]. Recent computational techniques using marker gene sets have enabled metagenomic sequencing approaches to be used for tracking microbial strains across different samples [13–15]. These methods track strains by first using available isolate references to pre-compute species-specific marker gene sets. Alignments of metagenomic short reads to these markers can then be used to estimate strain-specific single-nucleotide variant profiles, which can be tracked across samples. Although these approaches provide an efficient solution for the tracking of microbial strains, they are inherently limited to the study of clades with at least one representative isolate reference genome. Furthermore, these methods are unable to distinguish between very closely related strains sharing the same marker SNV profile, but differing in sequence from recent horizontal gene transfer or transposition events.

Dedicated metagenomic assemblers [16–18] and binning approaches based on sequence similarity [19–22] and coverage depth covariance [23, 24] aid analyses of metagenomic short-read sequences without relying on available genome references. These tools can yield significantly more comprehensive draft genomes, but have difficulty in resolving the correct genomic context of conserved and recently duplicated sequences, such as those arising from recent horizontal gene transfer and transposition. Culture-based methods can be helpful in this context, but require laborious and potentially biasing culture steps. In this respect, long-read sequencing platforms have been developed to address these drawbacks. However, these techniques typically require a 100- to a 1000-fold higher input of high-molecular-weight DNA, have lower throughput, and have a higher error rate when compared to short-read sequencing.

To address these challenges, we recently developed a metagenomic shotgun sequencing and assembly method that provides complete microbial genomes from complex microbiome samples [24]. The molecular approach of linked-read (or “read cloud”) library preparation relies on partitioning long DNA fragments within more than a million nanoliter droplets [25]. Barcoded short fragments are then derived with degenerate amplification of the long fragments within the partitions, pooled, and sequenced with Illumina technologies. Hence, the resulting “read clouds” are short-read sequences that contain long-range information [26].

In this case study, we present the application of read cloud sequencing to longitudinal microbiome samples from a hematopoietic cell transplantation (HCT) patient to demonstrate the evolution of gut microbes during therapy. This experimental approach enables the discovery of strain-level sequence variants that confer selective advantages under differing environmental stresses in a clinical setting. We find that exposure of the patient to antibiotics is correlated with the predominance of strains with specific transposon integrations that result in differential transcription of antibiotic resistance genes. In vitro antibiotic susceptibility testing and whole genome sequencing of isolates derived from the patient stool samples support functional predictions made using our metagenomic approach.

## Methods

### Sample collection

Stool samples were obtained from the study subject on an approximately biweekly basis, when available. Stool samples were placed at 4 °C immediately upon collection and processed for storage at − 80 °C the same day. Stool samples were aliquoted into 2-mL cryovial tubes with either no preservative or 700 μL of RNAlater and homogenized by brief vortexing. The aliquots were stored at − 80 °C until extraction.

### DNA library preparation

Stool DNA was extracted for short-read libraries and Gemcode read cloud libraries with the QiAMP Stool Mini Kit (Qiagen, Hilden, Germany) modified with an additional step after addition of ASL buffer consisting of 7 cycles of alternating 30-s periods of beating with 1.0 mm Zirconia/Silica beads in a Mini-Beadbeater (Biospec Products, Bartlesville, OK) and chilling on ice. Stool DNA was extracted for Chromium read cloud libraries with the Gentra Puregene Yeast/Bacteria kit (Qiagen, Hilden, Germany), modified with a chilling step at − 80 °C for 5 min, followed by ethanol DNA precipitation at 14,000*g* for 20 min at 4 °C.

Prior to read cloud sequencing library preparation, DNA was size-selected with the BluePippin instrument (Sage Science, Beverly, MA). A 5–50-kb size range was used for Gemcode libraries, and a 10–50-kb size range for Chromium libraries. Read cloud libraries were then prepared with either the Gemcode or Chromium instrument (10X Genomics, Pleasanton, CA).

DNA used for short-read library preparation was not size selected. Short-read libraries were prepared with the Truseq DNA HT library prep kit (Illumina, San Diego, CA).

All library fragment sizes were assessed with the 2100 Bioanalyzer instrument (Agilent Technologies, Santa Clara, CA) using the High Sensitivity DNA chip and reagent kit. DNA and library concentration estimations were performed using fluorometric quantitation with the Qubit 3.0 fluorometer using the Qubit dsDNA HS kit (Thermo Fisher Scientific, Waltham, MA).

### RNA library preparation

RNA was extracted with the Qiagen RNeasy Mini kit from stool samples stored in RNAlater at − 80 °C. Original total RNA concentration was assayed with the Qubit RNA HS kit. RNA was then ethanol precipitated and resuspended in nuclease-free water to concentrate, and then quantified again using both Qubit RNA HS and Qubit DNA HS kits to determine the degree of DNA contamination. Contaminating DNA was removed using the Baseline-ZERO DNase protocol (Epicenter, Madison, WI) with 30 min incubation followed by a second ethanol precipitation. Ribosomal RNA was depleted with the Epicenter Ribo-Zero rRNA removal kit (Bacteria) and purified with another ethanol precipitation. The rRNA-depleted mRNA quality was assessed with the 2100 Bioanalyzer using the Agilent RNA6000 Pico kit and quantified with Quibit RNA HS assay. cDNA sequencing libraries were then prepared with the Illumina Truseq Stranded mRNA kit following the Truseq Stranded mRNA LT protocol. Resulting DNA libraries were quantified with Qubit DNA HS kit and their quality evaluated by 2100 Bioanalyzer instrument using the High Sensitivity DNA chip and reagent kit.

### Sequencing

Truseq libraries were sequenced with 2 × 101 bp reads on an Illumina HiSeq 4000 instrument, each library receiving 2–6 Gb sequence coverage with the exception of timepoint A, which was additionally sequenced with 34 Gb of coverage after the initial attempt produced insufficient coverage. 10X Gemcode libraries were sequenced with 2 × 148 bp reads on an Illumina NextSeq 500 instrument, with each library receiving 4–7 Gb sequence coverage. 10X Chromium libraries were sequenced with 2 × 151 bp reads on one lane of Illumina HiSeq 4000, each receiving 18–22 Gb of sequence coverage. RNAseq libraries were sequenced with 2 × 101 bp reads on an Illumina HiSeq 4000 instrument, each library receiving 8–12 Gb sequence coverage with the exception of timepoint A, for which a high-quality RNA sequencing library could not be obtained. Total reads and sequencing coverage for all metagenomic sequencing libraries before and after quality control can be found in Additional file 1: Table S10. All raw sequencing data are available on SRA Bioproject accession PRJNA434731 [27].

### Metagenome assembly and genome draft generation

Raw reads from all DNA libraries were first subjected to the same quality control and trimming as follows. Sequence data were trimmed using cutadapt [28] using a minimum length of 60 bp and minimum terminal base score of 20. Reads were synced and orphans (reads whose pair mates were filtered out) were placed in a separate single-ended fastq file with an in-house script. RNA sequencing reads displayed uniform high quality and were not trimmed.

Trimmed reads from all libraries were then assembled using metaSPAdes 3.11.1 [29] with default parameters for paired-end input. MetaSPAdes seed assemblies obtained from read cloud libraries were then further assembled using Athena [30]. Assemblies were visualized with IGV [31], R [32], and python using the ggplot2 [33], circlize [34], and matplotlib [35] libraries.

For read cloud and short-read libraries, coverage was calculated for assembled contigs by aligning raw short reads with BWA v0.7.10 [36]. Metabat v2.12.1 [22] was then used to group contigs to form draft genomes. Drafts were then evaluated for a number of criteria to assess quality: Metaquast v4.6.0 [37] for N50 and assembly size, CheckM v1.0.7 [38] for genomic completeness and contamination, Prokka v1.12 [39] for gene counts, Aragorn v1.2.36 [40] for tRNA counts, and Barrnap v0.7 [41] for rRNA subunit counts. Drafts were denoted “high quality” when they contained 18 or more tRNA loci, at least one occurrence each of the 5S, 16S, and 23S ribosomal RNA subunits, and achieved a checkM score of at least 90% completeness and at most 5% contamination in accordance with existing standards [42]. Drafts were otherwise denoted “complete” if they achieved the same completeness and contamination criteria. All other drafts were denoted “incomplete.”

Individual contigs from all assemblies were assigned taxonomic classifications using k-mer-based classification with Kraken2 [43] with a custom database constructed containing all bacteria, viral and fungal genomes in NCBI GenBank assembled to complete genome, chromosome, or scaffold quality as of February 2019 [44, 45]. Human and mouse reference genomes were also included in the database. Genome drafts were given taxonomic assignments with a consensus approach: a draft received a species assignment if 60% or more of total bases shared the species-level classification. Drafts were otherwise assigned the majority genus-level classification.

### Discovery of genomic island integration and insertion sequence loci in read cloud metagenomic drafts

To discover large-scale genomic island incorporations, we first obtained pairwise sequence alignments of *Bacteroides caccae* genome drafts between timepoints (A, B), (B, C), and (C, D) using MUMMER [46]. We searched these alignments for instances in which a single contig from one timepoint produced a gapped alignment over a single contig from another timepoint, spanning a putative genomic island (Additional file 1: Fig. S3). Potential genomic island incorporations that were not assembled within a single contig could not be fully resolved and were not considered. No differential genomic island incorporations were observed in timepoint B with respect to timepoint A. Three separate genomic islands (17 kb, 57 kb, and 71 kb) were found in the *B. caccae* genome within timepoint C, but were absent from the draft in timepoint B. The 17 kb and 57 kb islands were also found within the draft genomes of *B. vulgatus* and *B. uniformis.* Two genomic island incorporations of sizes 43 kb and 50 kb were observed in timepoint D with respect to timepoint C. Neither of these two islands were assembled into alternate genomic contexts in timepoint C.

To discover smaller-scale insertion sequences, we examined high-frequency *k-mer* sequences that were present in the *B. caccae* genome. We first obtained *k*-mer counts from the timepoint C read cloud draft using Jellyfish [47] with *k* = 101. The vast majority of *k*-mers originate from single-copy portions of the genome, but we isolated the subset of these that occurred at a copy number of at least 10, and assembled them using SPAdes [48] with “--only-assembler” and “--sc”. This process yielded the sequences of two candidate insertion sequence elements within the *B. caccae* genome.

### Insertion sequence abundance estimation

Illumina Truseq short-read data were aligned with BWA [36] to the insertion sequence integration regions obtained from read cloud and Athena assembly of the clinical microbiome data. Reads recruited to each insertion locus were realigned with STAR [49] in order to obtain gapped alignments spanning the insertion sequence. Gapped alignments, representing the genome sequence prior to insertion (“ancestral strain”), were counted for each insertion. Ancestral strain fraction was expressed as the number of observed gapped alignments divided by the median sequence coverage within the neighboring 10 kb of sequence.

### *Bacteroides caccae* isolation

Members of the *Bacteroides fragilis* group, including *Bacteroides caccae*, were isolated directly from stool by streaking stool on solid BHI medium (containing 37 g/L brain heart infusion powder, 1% v/v Remel defibrinated sheep blood, 100 μg/mL gentamicin, and 1.5% agar) under anaerobic conditions in a Bactron 300 anaerobic chamber (Sheldon Manufacturing Inc., Cornelius, OR). Individual colonies matching the described *B. fragilis* group morphology of circular, entire, convex, gray, translucent, shiny, and smooth [50] were picked into 5 mL liquid tryptone yeast glucose (TYG) media [51] and incubated overnight inside the anaerobic chamber at 37 °C. Aliquots of liquid cultures with glycerol added to 30% final concentration were frozen at − 80 °C. Subsequent whole genome sequencing of DNA extracted from these liquid cultures was used to confirm the species identity of each isolate.

### Isolate sequencing, assembly, and annotation

We extracted DNA using the Qiagen Gentra Puregene Bacteria DNA kit and prepared Illumina Nextera XT short-read sequencing libraries from all of the isolates that were cultured from the stool samples. The resulting 53 sequencing libraries were multiplexed and sequenced on a single Illumina HiSeq 4000 lane (data on all isolate sequencing in Additional file 7: Table S9).

Short reads from each library were trimmed using the same procedure applied to stool sample libraries. Trimmed reads were then assembled using SPAdes [48] to obtain genome drafts, and contigs from each draft were taxonomically annotated using Kraken v0.10.6 [43] with a custom database constructed from the Refseq and Genbank [44, 45]. Genes were identified using Prokka v1.12 [39].

### Genotyping of IS614 and genomic island integration loci in *B. caccae* isolates

Alignments of the short reads back to their respective genome drafts allowed discovery of IS614 integration loci present in each *B. caccae* isolate genome. For each isolate, a modified genome draft was first obtained by masking sequences of partially reconstructed IS614 elements, and inserting a single fully assembled IS614 sequence. Raw reads were then mapped back to this modified genome draft using BWA [36]. Candidate flanking sequences downstream of the IS614 promoter in each isolate were then found by examination of spanning read pairs mapping between the added IS614 sequence and other sequence contigs. Isolates share flanking sequences as the majority of integrations occur more than once. These were reconciled to obtain a unique flanking set, and the IS614 genotype was determined for all isolates from this unique set.

In order to search for potential large genomic island sequences that are exclusively integrated into *B. caccae* isolate genomes of a particular timepoint, *k*-mer counts from all isolate genome drafts are first obtained using Jellyfish [47] with *k* = 31. For pairs of timepoints (A, C) and (C, D), we then searched for sets of *k*-mers that were exclusively present in the antecedent points, but not preceding ones. Each set of *k*-mers was then assembled using SPAdes [48] with “--only-assembler” and “--sc” to obtain candidate island sequences. This procedure yielded only two such large genomic islands, both of which were also found using comparisons of our read cloud *B. caccae* drafts.

### Antibiotic susceptibility testing and MIC determination

*B. caccae* isolate strains were first selected for testing on the basis of their IS614 genotype determined by whole genome sequencing. One isolate from timepoint A (A2) was determined to contain both *B. caccae* and another *Bacteroides* species most closely related to *Bacteroides uniformis*. A2 was subsequently re-streaked on BHI plates; this allowed for isolation of the *B. caccae* strain, which was verified using PCR (described below).

Susceptibility to ciprofloxacin and trimethoprim was assessed using a broth microdilution method for determining the minimum inhibitory concentration (MIC) adapted from CLSI [52] as follows. Ciprofloxacin was dissolved in acidified water (0.1 N HCl) to a final concentration of 25 mg/ml and trimethoprim was dissolved in DMSO to a final concentration of 50 mg/ml. -Culture tubes, TYG media, antibiotic stocks, 96-well assay plates, pipette tips and all other labware were reduced overnight in an anaerobic chamber prior to culture. Each strain was first grown to saturation directly from prepared glycerol stocks in 2.5 mL of TYG liquid culture for 48 h.

Clear 96-well flat-bottom assay plates were prepared with TYG media containing a twofold serial dilution of each drug over 11 concentrations in addition to no drug controls (ciprofloxacin range 0–4096 μg/ml; trimethoprim range 0–512 μg/ml). Ciprofloxacin precipitated at concentrations higher than 512 μg/ml after incubation period; thus, these concentrations were excluded from susceptibility analysis. The turbidity of the overnight cultures was determined by measuring the optical density at 600 nm (OD_600_), and the culture were adjusted with TYG media to normalize the densities across all strains. The cultures were diluted 1:200 in fresh TYG media (OD_600_ ~ 0.1) and set up in the assay plates (100 μL per well) that were already prepared with the drug dilutions (100 μL per well) for a final total volume of 200 μL per well. Each selected *B. caccae* isolate was tested in at least two and up to four replicates. Assay plates were incubated for 48 h at 37 °C in the anaerobic chamber. The OD_600_ was then measured for each plate on a BioTek Epoch spectrophotometer (BioTek Instruments, Inc., Winooski, VT). Prior to plate reading, wells were mixed by pipetting up and down to evenly mix the cultures as all *B. caccae* strains were observed to flocculate over the incubation period. The minimum inhibitory concentration (MIC) of each drug for each replicate of each isolate was determined by fitting the drug concentration vs. OD_600_ data to a Gompertz function [53]. The MIC values were then rounded to the nearest discreet MIC category (32–64–128–256–512 μg/ml). The differences in resistance phenotypes were evaluated using a Student *t*-test with a significance threshold of *p* value ≤ 0.05.

### PCR amplification

PCR was performed to verify insertion sequence presence across timepoints at select loci where they were found to be assembled. PCR reactions contained Phusion High-Fidelity DNA Polymerase (New England BioLabs, Ipswich, MA) with Phusion HF Buffer and NEB Deoxynucleotide Solution Mix. Primers were obtained from Elim Biopharm (Hayward, CA) with target melting temperature of 60 °C. Reactions consisted of 6 μL 5X Phusion HF buffer, 0.6 μL 10 mM dNTP, 0.3 μL Phusion, 2 μL 10 mM forward primer, 2 μL 10 mM reverse primer, 1 μL template DNA, and PCR clean water to 30 μL. Thermocycling was performed with 30 s of denaturation at 98 °C followed by 35 cycles of 5 s of denaturation at 98 °C, 10 s of annealing at 65 °C, and 30 s per kilobase extension at 72 °C. This was followed by a final extension at 72 °C for 5 min and an indefinite hold at 4 °C.

## Results

### Clinical findings

**Fig. 1:**
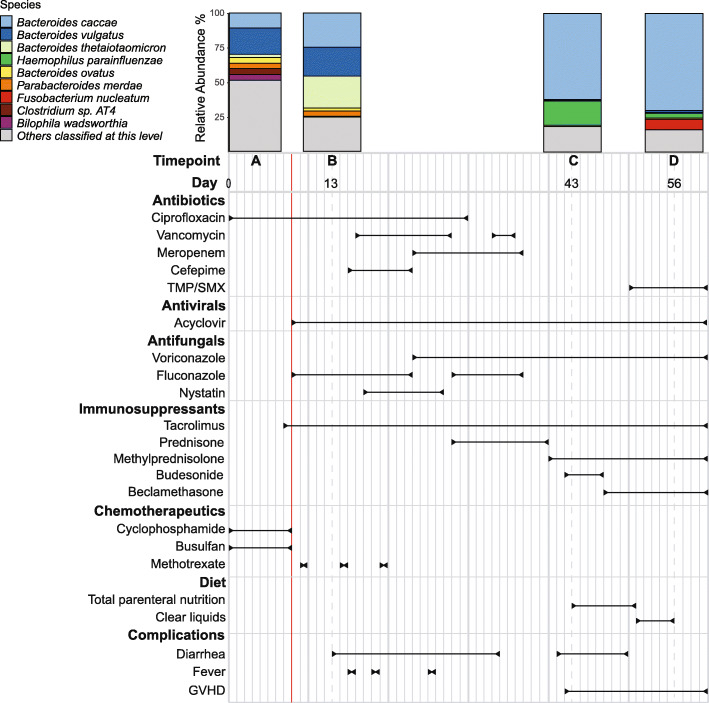
Patient condition, drug exposure, and intestinal microbiome composition during treatment. The study subject was admitted to Stanford Hospital with myelodysplastic syndrome and myelofibrosis and subsequently underwent hematopoietic cell transplantation (HCT, denoted by a red line). Stool samples were collected prior to HCT and over the following 5 weeks as the patient underwent chemotherapy, antibiotic treatment, and immunosuppression. Taxonomic classification of shotgun metagenomics sequencing reads (Illumina TruSeq Nano DNA) reveals pronounced dysbiosis emerging following HCT with gut domination by *Bacteroides caccae*, a commensal bacterium. Relative abundances of each species are determined using reads that are classified at the species level. The relative abundance of the species in the samples was determined after removing human and viral reads from the data. For this figure, the top 9 most abundant species in the shotgun metagenomics data are shown. Relative abundance data for all the species-level classification is shown in Additional file 2: Table S1. For a more detailed view of the species-level and genus-level classifications of all the samples, refer to Additional file 1: Fig. S1

The patient was a 46-year-old man who underwent HCT at the Stanford Hospital for treatment of an underlying myelodysplastic syndrome and myelofibrosis, which was refractory to treatment with azacitidine. The patient had been exposed to a course of therapy with azithromycin 1.5 months prior to the date of the first sample (Fig. 1—timepoint A); treatment with ciprofloxacin started the day before the first sample was taken. Treatment with two cycles of azacitidine treatment took place 2.5 months and ~ 1 month prior to timepoint A. The patient received multiple medications during the period of observation including antibacterials, antivirals, antifungals, chemotherapy, and immunosuppression (Fig. 1). The patient underwent colonoscopy and was diagnosed with stage II gastrointestinal (GI) graft-versus-host disease (GVHD) on day 42 after transplantation. He received immunosuppression (tacrolimus, prednisone, methylprednisolone, budesonide, and beclomethasone) between days 7 and 60 as prophylaxis and treatment for GVHD. The patient was provided a standard neutropenic diet until engraftment. On day 43, the patient’s diet was restricted to total parenteral nutrition for 8 days before he was advanced to a clear liquid diet. Stool samples were collected from the patient over the course of 56 days.

### Oligo-domination of the intestinal microbiome during treatment

To study the trajectory of this patient’s intestinal microbiome throughout treatment, we selected the following four timepoints for sequencing: A (day 0), pre-chemotherapy and pre-HCT; B (day 13), post-chemotherapy and post-HCT; C (day 43), post broad-spectrum antibiotic exposure and onset of GI GVHD; D (day 56), following onset of GVHD and introduction of a new antibiotic regimen including trimethoprim (Fig. 1). We note that the first sample we collected for this patient 1 day after the patient was exposed to ciprofloxacin; he had also been exposed to a course of therapy with azithromycin 1.5 months prior to the date of the first sample. Thus, our study lacks a sample taken prior to antibiotic treatment.

We applied read cloud sequencing to the four clinical stool samples obtained from the patient. Read cloud and standard short-read sequencing libraries were prepared from DNA extracted from each of the samples. Additionally, short-read libraries were prepared from RNA extracted from each of the samples (see “Methods”). Species-level community compositions obtained by both short-read and read cloud approaches were first assessed using *k*-mer-based short-read classifications (Additional file 1: Fig. S1).

During the course of treatment, the patient’s intestinal microbiome underwent profound simplification, rapidly becoming dominated by *Bacteroides caccae*, a normal resident within a healthy human intestinal microbiome [54] (Fig. 1; genus-level classifications included in Additional file 1: Fig. S1). Both sequencing approaches displayed domination by *B. caccae* in later timepoints C and D, but differed significantly in community composition for earlier timepoints A and B. Read cloud libraries had a higher relative abundance of Gram-positive bacteria versus Gram-negative bacteria in these timepoints as compared to short-read libraries. This discrepancy may be the result of differences between the lysis methods used to extract DNA for read cloud and short-read libraries [55, 56] (see Additional file 1: Supplementary Results).

### Read clouds produce more contiguous and complete genome drafts of *B. caccae*

To obtain individual genome drafts for constituent microbes present in each sample, short-read and read cloud libraries were first assembled using either a conventional short-read assembler alone or a short-read assembler and Athena [25], respectively (see “Methods”). Contigs in each metagenome draft were then binned and taxonomically classified to obtain annotated genome drafts (see “Methods”). CheckM [38] was applied to the resulting bins to assess genome completeness and contamination by the presence of lineage-specific single-copy core genes. In addition to assembling *B. caccae* genome drafts, read clouds produced well-assembled genome drafts for other *Bacteroides* members *Bacteroides vulgatus* and *Bacteroides uniformis* (Additional file 4: Table S3). For timepoints A and B, read clouds also assembled several high-quality genome drafts [42] with N50 values exceeding 500 kb for members of enriched Gram-positive genera including *Eubacterium*, *Lachnospiraceae*, *Gemella*, and *Flavonifractor*. Read clouds produced single genome bins for *B. caccae* that were 94% complete and < 1% contaminated in dominated timepoints C and D, but produced multiple, less complete bins for *B. caccae* in earlier timepoints A and B (Additional file 5: Table S4).

**Fig. 2:**
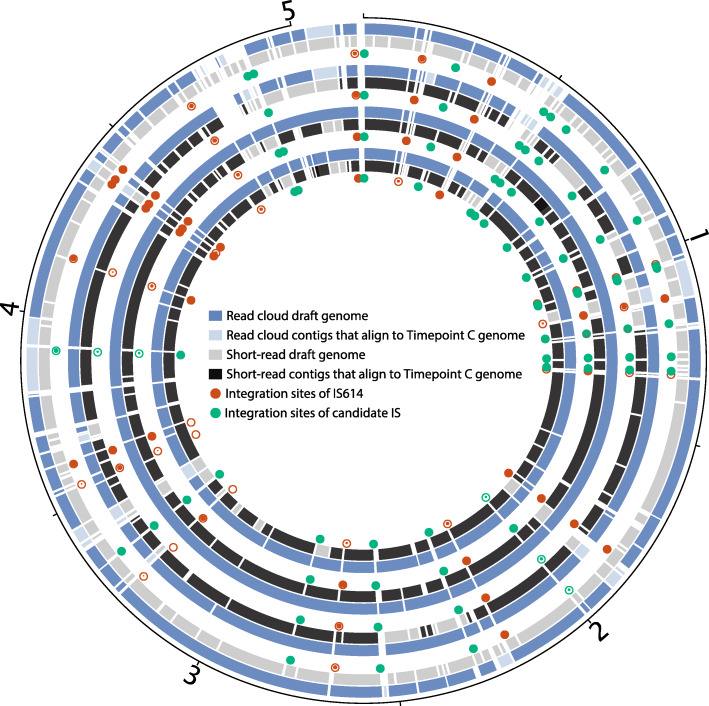
Read cloud and short-read genome drafts of Bacteroides caccae obtained from sequencing of stool samples. Circos plots of draft genome assemblies of *B. caccae* from the sequencing of the patient stool samples are ordered chronologically (A through D) from the outermost inward. Read cloud genome drafts assembled using Athena (blue) are more contiguous and complete than short-read drafts assembled using conventional assembly (gray). The read cloud *B. caccae* draft from timepoint C (third circos plot from the outside) was the most contiguous and served as the reference for all alignments. Contigs from each read cloud and short-read library are assigned a lighter color if they aligned to this reference, but did not belong to a draft classified as *B. caccae*. Short reads did not produce a draft annotated as *B. caccae* for timepoint A. The best read cloud and short-read drafts are both obtained through sequencing of timepoint C (read cloud: 414 kb N50, 5.5 Mb size, 99.3% complete, 1.6% contaminated; short read: 88 kb N50, 4.7 Mb size, 97.7% complete, 0.6% contaminated). The read cloud drafts include a total of 18 assembled integration sites of IS614 (red circles) and 25 assembled integration sites of a candidate insertion sequence (green circles) that are missing from all short-read drafts. Alignments of raw short reads to these sites indicated the presence of both strains without insertion sequence integration and strains with the insertion sequence integration. Estimated proportions of these strains for each site and timepoint are shown with different filled in areas of each circle, with an empty circle denoting predominance of ancestral strains lacking an IS at that location and shaded circles denoting predominance of strains with the IS at that location

To produce more complete drafts for *B. caccae*, bins annotated as *B. caccae* in each assembly were merged and reevaluated using checkM as more complete genome drafts (Additional file 1: Table S5). Read clouds with Athena assembly were able to consistently yield more contiguous and more complete drafts of *B. caccae* than short-read sequencing and assembly (Fig. 2). Despite differences in overall sequence coverage and community composition, *B. caccae* had comparable absolute raw sequence coverage in both short-read and read cloud libraries (Additional file 1: Table S5). Our most contiguous and complete *B. caccae* read cloud draft was from timepoint C with an N50 of 414 kb and total size of 5.5 Mb; this is a notable improvement over the best short-read draft, which was from timepoint C with an N50 of 88 kb and total size of 4.7 Mb. *B. caccae* coverage varied between 27× and 1542× across read cloud libraries, and 157× and 759× across short-read libraries. All four *B. caccae* read cloud drafts showed large-scale structural concordance with the available closed reference genome (Genbank ID GCA_002222615.2), with the exception of one misassembly in the timepoint D draft around a 16S/23S ribosomal RNA gene operon (Additional file 1: Fig. S2).

We next compared the best read cloud and short-read *B. caccae* drafts, which were both produced in libraries from timepoint C, to available reference isolate genomes. We first aligned the six available reference isolate genomes of *B. caccae* (Genbank IDs NZ_AAVM02000021.1, NZ_JH724079.1, NZ_CZBL01000001.1, NZ_CZAI01000001.1, NZ_CP022412.2, NZ_PUEQ01000001.1) to our read cloud drafts using MUMMER [46]. The read cloud *B. caccae* genome contained 639 kb of novel sequence not represented in any reference isolate, compared to just 318 kb of novel sequence contained in the short-read draft. The median sequence identity for alignable bases between our *B. caccae* drafts and the reference isolates was 99.5% for both read clouds and short reads (Additional file 1: Table S5).

### Closely related but divergent strains coexist in clinical samples

We posited that comparative analysis of the *B. caccae* genome drafts across timepoints would provide insight into either strain selection or potential genomic remodeling of this organism as it grew to eventually dominate the host’s intestinal microbiome. We first searched the read cloud *B. caccae* drafts for differing large-scale genomic island incorporations. Pairwise alignments of *B. caccae* drafts from successive timepoints were first obtained with MUMMER [46], and we used these alignments to identify large genomic sequences that were assembled into different genomic contexts between drafts from different timepoints (see “Methods”, Additional file 1: Fig. S3). We identified a total of five separate genomic islands ranging from 17 to 71 kb in size that were integrated into different genomic contexts between drafts of different timepoints (Additional file 1: Table S6). Two of these islands were also present in the draft genomes of *B. vulgatus* and *B. uniformis*.

We next searched the read cloud *B. caccae* drafts for small-scale insertion sequences. Insertion sequence elements were first identified by counting *k*-mers in each read cloud *B. caccae* draft, selecting high-frequency *k*-mers, and assembling these to determine their precise sequence (see “Methods”). This procedure yielded two putative insertion sequence elements. The first IS, 1596 bp in length, was annotated using nucleotide alignments with BLAST (nt database) [57]. It was determined to be IS614, a conserved Bacteroides insertion sequence (IS). IS614 encodes a transposase as well as an outward-facing promoter sequence, which has been predicted to drive transcription of genes neighboring the IS [7]. The second IS, 1470 bp in length, could not be annotated as a previously described IS, but shares protein sequence homology and a conserved DDE domain with the IS4 insertion sequence family. Both IS614 and the unannotated candidate IS appear in the metagenome drafts of the short-read libraries, but appear only in single copies detached from genomic context with extreme sequence coverage depth (16,664× and 14,615× coverage for IS614 and the candidate IS in timepoint C, respectively). This suggests that high copy sequence elements, including these insertion sequences, cannot be assembled with short-read assembly, greatly limiting the overall quality of genome drafts obtainable with this approach. Although we found the candidate IS to be present within two reference isolate genomes for *B. caccae* (Genbank IDs: NZ_CZBL01000001.1, NZ_CZAI01000001.1), we could not identify IS614 in any of the six available reference isolate genomes.

We searched for integrations of IS614 and the candidate IS across all read cloud metagenome drafts in which at least 3 kb of flanking sequence was assembled on both sides of the integration. We found 28 unique integrations of IS614, 18 of which were determined to be in *B. caccae*, and 10 in *B. vulgatus*. We found 25 unique integrations of the unassigned IS, all of which were determined to be in *B. caccae*.

**Fig. 3:**
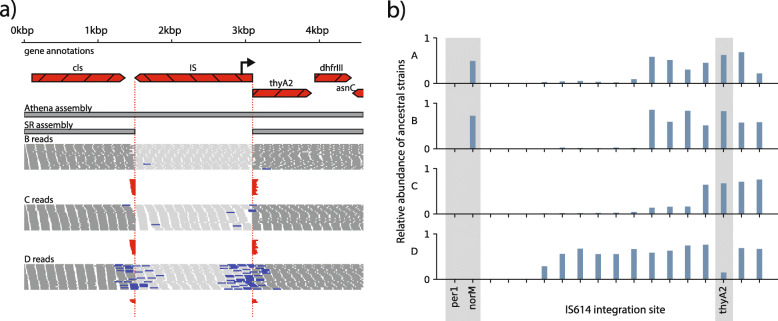
Co-occurrence of multiple *B. caccae* strains with differing IS614 integrations. **a** Alignments of short reads from timepoints B, C, and D to a representative IS integration site reveal domination of the strain without the IS (“ancestral strain”) in timepoints in B and C, and domination of the strain harboring the insertion in D. Short-read alignments from B and C show many reads spanning over both left and right junctions (red), indicating global alignment to the ancestral sequence, while short-read alignments from D show many reads supporting the IS integration (blue), indicating read pairs or single reads spanning the IS. This demonstrates that the IS is present at this locus at timepoint D but is undetectable at timepoints B and C. **b** Estimated relative abundances of *B. caccae* ancestral strains and strains with an IS integration for all 18 detected IS614 integration sites. Integrations upstream of annotated antibiotic resistance genes *norM*, *thyA2*, and *per1* are shaded. Major shifts in abundances amongst the strains with and without integrations upstream of *norM* and *thyA2* can be seen between timepoints B and C and timepoints C and D, respectively. Integration sites are sorted by ancestral strain fraction in timepoint C

At many insertion sites in *B. caccae*, alignments of the short-read data to the Athena assembly confirmed the co-occurrence of both strains harboring the IS and strains with the pre-insertion “ancestral” sequence (Fig. 3a). From these alignments, we obtained an estimate of the relative abundance of ancestral and insertion-containing strains for each site (see “Methods”). Only three of the 25 identified integration sites of the candidate IS had short-read alignments indicative of a pre-insertion ancestral sequence. The rest of these integrations appeared to be fixed in the *B. caccae* strain population. By contrast, we observed large shifts in ancestral abundance at several *B. caccae* IS614 integration loci (Fig. 3b), with 15 large (> 30%) shifts occurring between consecutive timepoints. Additional file 1: Table S7 summarizes the fractional abundances of ancestral strains at each assembled *B. caccae* insertion site.

### Insertion sequences mediate transcriptional upregulation in *B. caccae*

**Fig. 4:**
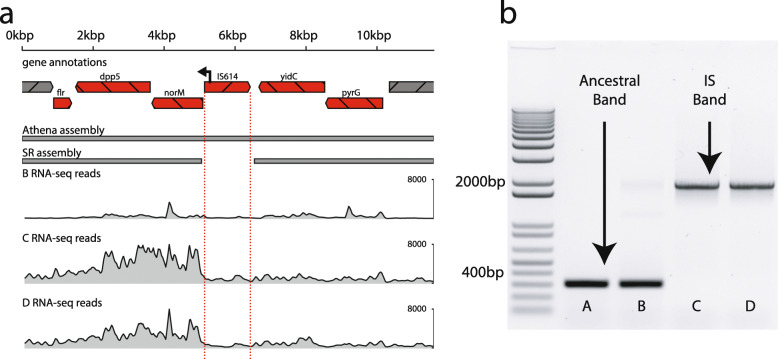
Metatranscriptomics support IS-mediated transcription within *B. caccae.* IS614 contains a putative outward-facing promoter. The relative contribution of the IS promoter to transcription was determined by comparing RNA sequencing read depths of genes upstream and downstream of it. **a** In timepoint B, which is dominated by ancestral strains without the promoter, RNA sequencing read coverage depth (relative transcript abundance) is relatively equal on both sides of the integration site. In timepoint C, which is dominated by strains with IS614 with its putative outwardly directed promoter, the transcription of the downstream gene *norM* is much higher than that of the upstream gene *yidC*. The relative transcript abundance of all neighboring genes increase in timepoint C relative to B, but this increase is 10-fold greater in genes immediately downstream of the introduced outward promoter. In later timepoints C and D, dominant strains harbor an introduced IS promoter positioned to upregulate *norM*. This is supported by read pairs spanning between the IS promoter and *norM*. This difference in coverage and domination by strains with this promoter both persist through timepoint D. Conversely, the earlier timepoint B is dominated by strains with no IS in this region. **b** PCR with primers flanking the above integration instance of IS614 yields amplicons without the insertion sequence in earlier timepoints A and B (400 bp), and with the insertion in later timepoints C and D (1.9 kb)

We next used RNA sequencing of each timepoint to investigate the potential transcriptional effects of the genomic alterations we detected in *B. caccae*, focusing on IS614. This IS contains a putative outward-facing promoter near its 5′ end oriented antisense to its transposase coding sequence [7]. Determining the transcriptional effect of this outward-facing promoter is difficult in a complex metagenomic setting, as RNA sequencing reads may originate from co-occurring strains with or without a given insertion. In light of this difficulty, we restricted our attention to integration sites that were dominated first by ancestral strains and then by IS-harboring strains in consecutive timepoints, with at least 30% change in estimated ancestral abundance. The 30% threshold was selected because it is the minimum change in ancestral strain abundance that was needed to unambiguously assign RNA sequencing reads to the correct strain in this complex metagenomic mixture. In these sites, a corresponding increase in transcription of genes downstream of the promoter versus upstream of the promoter is more likely attributable to the additional promoter introduced by the IS. We found five such candidate loci, four of which had putative promoter sites introduced in the same sense as the adjacent downstream gene. All four of these loci showed “transcriptional asymmetry” in which transcription of the downstream neighboring gene was increased between threefold and eightfold relative to transcription of the upstream neighboring gene. This coincided with an increase in relative abundance of strains harboring the insertion (Fig. 4, Additional file 6: Table S8).

One notable transcriptional asymmetry coincided with placement of the putative promoter in IS614 to upregulate *norM*, a multidrug resistance transporter (Fig. 4a). *NorM* is a multidrug efflux protein that can confer mild resistance to ciprofloxacin [58, 59]. Ciprofloxacin was administered for the first 30 days of treatment through timepoints A and B (Fig. 1). Manual inspection of short-read alignments to this insertion site showed this integration to be undetectable in timepoint A, present in roughly a third of strains in B, and then in the majority in timepoints C and D, consistent with visible band patterns in our targeted PCR results (Fig. 4b, Additional file 6: Table S8). Another transcriptional asymmetry was observed adjacent to *thyA2* and *dhfrIII*, encoding thymidylate synthetase and dihydrofolate reductase, respectively (Additional file 1: Fig. S4). Both are linked to trimethoprim sensitivity [60, 61], and the marked rise in strains carrying an adjacent IS-borne promoter coincides with the administration of this antibiotic to the patient prior to the final timepoint D. A third transcriptional asymmetry was found in resA, an oxidoreductase involved in cytochrome c synthesis [62] (Additional file 1: Fig. S5). The abrupt changes observed in the abundance of insertion sequences adjacent to these loci suggest selective pressures applied to the bacterial strains.

We found the most highly expressed gene in timepoints C and D to be the extended-spectrum beta-lactamase gene *per1*, known to confer resistance to beta lactam antibiotics [63], which were administered to the patient between timepoints B and C. *Per1* was expressed nearly 60% more than the second most expressed gene in both timepoints C and D (see Additional file 1: Supplementary Results).

Though several insertion sequences became undetectable in DNA sequence data between timepoints C and D, strains with insertions adjacent to *norM* and *per1* continue to dominate through the end of the investigated time course. By timepoint D, ciprofloxacin and meropenem have been withdrawn for 26 and 19 days, respectively, yet expression of resistance genes *norM* and *per1* remained elevated compared to their levels prior to antibiotic exposure.

### Antibiotic susceptibility of *B. caccae* clinical isolates

Based on the analysis of the genome drafts and IS-mediated upregulation of antibiotic resistance genes in *B. caccae*, we hypothesized that strains from different clinical timepoints will show varying degrees in antibiotic susceptibility. To assess these phenotypic predictions, we isolated *B. caccae* strains from stool samples for whole genome sequencing and antibiotic susceptibility testing. Stool samples from each of the four timepoints A, B, C, and D were streaked directly on selective media to isolate members from the *Bacteroides fragilis* group including *B. caccae* (see “Methods”). A total of 53 colonies from timepoints A, C, and D were selected based on morphology and further cultured in liquid media. We were unable to obtain colonies representative of the *B. fragilis* group from the timepoint B stool sample. The isolates were sequenced and assembled to obtain draft genomes (see “Methods”, Additional file 7: S9).

Analysis of the assembled genomes revealed that 12 of the 53 isolates were not *B. caccae* (Additional file 7: Table S9). Notably, 7 out of 10 isolates from timepoint A belonged to a *Bacteroides* species closest to *Bacteroides uniformis*. Our isolate collection contained a total of 41 *B. caccae* strains with 3, 17, and 21 *B. caccae* strains from timepoints A, C, and D, respectively.

We examined sequencing data from the 41 *B. caccae* isolates to identify genomic alterations that may be under selection between different timepoints over the course of the patient’s treatment. We first detected the set of all flanking genomic sequences that were downstream of the putative bacterial promoter present in IS614, and also larger genomic islands that were differentially present between the isolates (see “Methods”). Each *B. caccae* isolate was then genotyped for the presence or absence of each IS614 integration and genomic island incorporation.

**Fig. 5:**
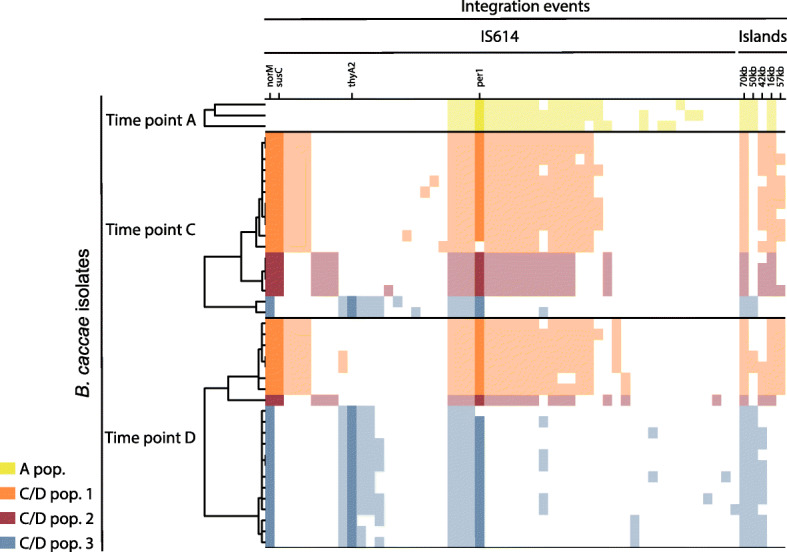
Genomic alterations detected in *B. caccae* isolates support selection throughout treatment. The detected proportions of IS614 and large-scale genomic island integrations (columns) in 41 *B. caccae* isolates (rows) are shown (filled squares indicate presence). Hierarchical clustering of the 41 isolates by their IS614 integrations within each timepoint reveals four distinct subpopulations of *B. caccae* strains, three of which appear to shift in relative abundance between timepoints C and D. The IS614 integration upstream of *norM* is the only one that is absent from all isolates from timepoint A (before ciprofloxacin exposure), but appears in all isolates from timepoint C and D (after ciprofloxacin exposure). The IS614 integration upstream of *per1* is present in all of the *B. caccae* isolates we obtained. Initial analysis of the isolate sequencing data was unable to detect IS614 integrations in front of *per1* for two isolates, but manual inspection of the assembly graphs of these confirmed this integration to be present in these two as well (open squares in the *per1* column). The IS614 integration upstream of *thyA2* appears in a minority of strains in timepoint C (prior to trimethoprim exposure) and appears in the majority of strains in timepoint D (after trimethoprim exposure). The IS614 integration upstream of *susC*, which was detected in isolate sequence data, also appears to be under selection in timepoint C. Unlike the IS614 integrations, none of the large-scale genomic islands appear to be under selection between timepoints as they are broadly distributed across all subpopulations

Four IS614 integrations were present in all *B. caccae* isolates, including *per1*, which we predicted to be fixed in the *B. caccae* strain population across timepoints using our read cloud metagenomic approach. Hierarchical clustering of *B. caccae* isolates by IS614 integrations revealed the presence of four distinct strain subpopulations that shift in abundance between timepoints (Fig. 5). Isolate sequencing also revealed an additional IS614 integration that was absent from our original read cloud assemblies and located upstream of the gene *susC*, a surface-accessible protein involved in starch binding and utilization [64]. This IS614 instance was present in high abundance in the latter timepoint isolates (15 out of 17 timepoint C isolates, 8 out of 21 timepoint D isolates), concordant with the time period of poor oral dietary intake and initiation of total parenteral nutrition.

All strains from timepoints C and D (post ciprofloxacin exposure) contain an IS614 integration upstream of *norM*, which is absent from all three strains from timepoint A (pre ciprofloxacin exposure). Although a few strains with an IS614 integration upstream of *thyA2* and *dhfrIII* are present in timepoint C (pre trimethoprim exposure), the majority of strains with this integration are present in timepoint D (post trimethoprim exposure). The observed shifts in abundance of the *B. caccae* strain subpopulations are consistent with the differing selective pressures applied from the patient’s drug regimen over the course of treatment. Notably, while clear subpopulations with varying IS614 positions appear to exist and have a selective fitness advantage at various timepoints, the larger genomic islands appear to be sporadically integrated amongst the individual strains. This suggests that none of the larger genomic islands were under selection between timepoints A and C or between timepoints C and D.

**Fig. 6:**
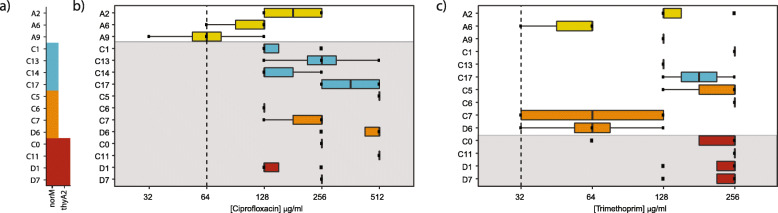
Antibiotic susceptibility testing of *B. caccae* isolates. Fifteen *B. caccae* isolates (3 isolates from timepoint A and 4 from each subpopulation present in timepoints C and D) were tested for their susceptibility to ciprofloxacin and trimethoprim, the two antibiotics that the patient was administered during treatment. The minimum inhibitory concentrations (MICs) of the two drugs against each isolate were determined as described in the “Methods”. **a** The presence (filled square) of different IS614 integrations within tested isolate strains that are upstream of annotated genes *norM* and *thyA2*, which are known to contribute to resistance to ciprofloxacin and trimethoprim. *NorM* is a known multidrug efflux pump that can confer resistance to ciprofloxacin. Upregulation of *thyA2*/*dhfrIII* has been shown to affect resistance to trimethoprim [61]. These IS integrations result in the potential introduction of likely bacterial promoters upstream of these genes likely leading to their upregulation increased expression and consequently as a result, increased antibiotic resistance. **b** MICs of ciprofloxacin against *B. caccae* strains. Strains from timepoints C and D with IS614 integrations upstream of *norM* and *thyA2/dhfrIII* predicted to increase resistance to ciprofloxacin (shaded) have a two- to fourfold increase in their MICs relative to strains from timepoint A. **c** MICs of trimethoprim against *B. caccae* strains. Overall, strains from all subpopulations showed high resistance to trimethoprim. Strains from the third subpopulation in timepoints C and D with IS614 integrations predicted to increase resistance to trimethoprim (shaded) show a twofold increase in resistance to trimethoprim compared to other subpopulations

We posited that the strain-level variants we identified will result in different drug resistance phenotypes. To address this hypothesis, we tested the susceptibility of several representative strains from each *B. caccae* subpopulation to ciprofloxacin and trimethoprim. In total, we selected all three strains from timepoint A and four strains from each of the three subpopulations of timepoints C and D for this analysis (Fig. 6a). The growth of each isolate was evaluated against a twofold serial dilution of each drug (see “Methods”). Growth was determined by measuring the optical density at 600 nm (OD_600_) (Additional file 1: Fig. S6, S7).

Overall, our antibiotic susceptibility analysis revealed that these clinical *B. caccae* isolates are more resistant to both ciprofloxacin and trimethoprim when compared to other members of the *B. fragilis* group (Fig. 6). Previously reported MIC_50_ values (antibiotic concentration at 50% growth inhibition) for members of the *B. fragilis* group have been ≤ 8 μg/mL for ciprofloxacin [65, 66] and ≤ 8.8 μg/mL for trimethoprim [67]. By contrast, the MIC_50_ values across all strain subpopulations were ≥ 64 μg/mL for both ciprofloxacin and trimethoprim. Nonetheless, we observed varying degrees of drug resistance in the isolated strains from the four subpopulations. Isolates from timepoints C and D, which we expected to be more resistant to ciprofloxacin using our metagenomic approach, showed a twofold to fourfold increase in the MICs relative to those from timepoint A (Fig. 6b). Isolates from the third subpopulation present in timepoints C and D, which we expected to be more resistant to trimethoprim based on our metagenomics analysis, showed a modest increase (twofold) in MIC compared to isolates from the other subpopulations (Fig. 6c).

Grouping of replicates by subpopulation assignment enabled the examination of their collective antibiotic susceptibility. We found that the difference in ciprofloxacin susceptibility between strains from subpopulation A and each of the three C/D subpopulations (Student *t*-test *p* value of 0.007, 0.0003, and 0.0009 for subpopulations C/D-1, C/D-2, and C/D-3, respectively). This suggests that the resistance phenotype to this drug appeared and was sustained over time. Trimethoprim resistance is consistently highest in the subpopulation with strains carrying the IS614 integration upstream of *thyA2*, although it is notable that some of the strains at timepoint C also have a high level of resistance in the absence of the IS614 integration upstream of *thyA2*. Differences in trimethoprim susceptibilities were statistically significant when comparing the MIC values of subpopulation A and subpopulation C/D-3 (*p* value = 0.001), and subpopulation C/D-2 and subpopulation C/D-3 (*p* value = 0.009). Collectively, the drug susceptibility results are generally consistent with the resistance phenotypes we predicted based on our metagenomics analysis. Whole genome sequencing of *B. caccae* isolate strains from timepoints A, C, and D together with antibiotic susceptibility testing support the presence of selection amongst strain populations of *B. caccae* with different IS614 integrations as predicted using our read cloud metagenomic approach.

## Discussion

Strain variation within microbial communities can arise either from de novo evolution or through selection upon standing variation. Tracking strain-level variation in the gut microbiome in a clinical setting remains an important challenge to understand the functional role of this variation in the clinic. Although computational approaches have allowed us to detect changes in genus-level and even species-level microbial community composition, it has remained difficult to track changes in the composition of strains that harbor more fine-grained structural differences.

Here, we leverage a recently developed read cloud metagenomic sequencing approach to create de novo genomes of bacterial strains from a case study of a clinical microbiome time series, which we then use to discover strain-level dynamics over the course of the patient treatment. The clinical subject we examine in this study underwent extensive treatment with several classes of medication while undergoing hematopoietic cell transplantation. Immune suppression and extensive antibiotic treatment likely contributed to destabilization of the intestinal microbiome. In other studies, this type of less-diverse intestinal microbiota has been associated with increased overall mortality, GI GVHD, and other adverse outcomes [68–71]. Previous studies of similar cohorts as well as healthy individuals have shown that antibiotic use can lead to intestinal domination by one or few microorganisms [72, 73], but the mechanisms by which any specific microorganism achieves dominance remain incompletely understood. Our study of this patient revealed that populations of microbes with apparently stable taxonomic composition can, in actuality, be composed of many closely related strains that undergo large fluctuations in abundance. The generalizability of this observation has yet to be evaluated and requires further investigation.

Using the read cloud sequencing and assembly approach, we observe large shifts in abundance of bacterial strains harboring different IS614 integration sites. Integration events are often biased to transcriptional hotspots where an open DNA conformation promotes higher rates of transposition with a transposon. Interestingly, we have only observed the IS element in one orientation whereas if the IS entry was secondary to very active transcription, we would expect an equal likelihood of both orientations of the IS element. Through RNA sequencing, we find evidence of gene upregulation caused by insertion sequence-mediated transcription. One IS614 integration site positioned to upregulate *norM* was found to be undetectable in *B. caccae* strains within timepoint A, present in a fraction of strains within timepoint B, and present in all detectable strains within timepoints C and D. This rise to dominance is consistent with both the observed timing of ciprofloxacin administration and the role of *norM* in antibiotic resistance [58]. Another IS614 integration site positioned to upregulate *thyA2* and *dfhrIII* is detected in strains at low abundance within timepoint C and present in the majority of detectable strains in timepoint D. This observed shift in strain abundance is again consistent with trimethoprim administration between timepoints C and D, as well as previous reports linking increased expression of these genes to trimethoprim resistance [60, 61]. Examination of isolate sequencing data revealed an additional integration positioned to upregulate *susC*, a gene involved in starch binding and utilization [64]. In light of the restrictions placed on the patient’s diet starting after timepoint B, it is possible that upregulation of *susC* rendered organisms with this selective advantage more successful in competing for limited starches available within the gut lumen. Our results suggest that insertion sequences can mediate changes in gene transcription within individual strains, which creates a pool of phenotypic variation that may allow adaptation to changing environmental stresses.

Our results highlight the value of de novo characterization of microbial communities in capturing strain-level variation, as well as the importance of strain dynamics in antibiotic-associated dysbiosis of the gut microbiome. Previous methods to detect genomic strain diversity rely on compiled reference sequence collections, characterizing nucleotide divergence within either predefined gene sets or gene presence within a predefined organism-specific pan-genome [74–76]. Although these methods differ substantially, their reliance on the reference sequence collection restricts sensitivity in the context of poorly sequenced or unknown species which comprise a large fraction of microbial diversity [77]. We note that for this dataset, genome assembly was relatively easier given the successive decrease in the diversity of the microbial community and eventual sweep by *B. caccae* in the samples. With the longitudinal data, we were capable of contextualizing the strain diversity of *B. caccae*. Our experience is in line with how community composition, genetic diversity, and other factors contribute to genome assemblies [78]. Existing short-read methodologies fail to assemble certain classes of sequences, including insertion sequences and larger-scale genomic island incorporations, both of which can impart significant changes to bacterial phenotypes. Read cloud assemblies can also be limited in resolving genomes with a high degree of sequence repetition. These repetitive insertion sequences often co-locate with breaks in both short reads and read cloud genome assemblies. We have encountered these challenges in obtaining complete genome assemblies of *Prevotella copri* using read clouds, and in these cases have ultimately used nanopore long-read sequencing and assembly to overcome these limitations [79].

Our results demonstrate that high-quality individual genome drafts may facilitate a better understanding of the complex interactions between strains and species within the microbiome. Further investigation is needed to determine if the rise in abundance of strains carrying different insertion sequence integrations is due to selection or an active biological process occurring in response to environmental stress. Although strains carrying the IS614 integration upstream of *norM* are undetectable by read cloud assembly or PCR in timepoint A before ciprofloxacin administration, it is possible that these strains were present at very low abundance and grew to dominate in later timepoints due to selection. Further in vitro adaptive evolution experiments may be able to determine whether IS614 is capable of mobilizing in response to the types of exposures and clinical situations faced by the studied subject. Whole genome sequencing of *B. caccae* strains isolated directly from the patient’s stool samples indicates the presence of four major strain subpopulations. Three of the four subpopulations contain an IS614 integration adjacent to *norM*, suggesting that these integrations arose from multiple independent transposition events. However, a higher number of isolate genomes from earlier timepoints and examination of more individual patients would provide more compelling evidence to support this hypothesis.

As methods for read cloud and long-read sequencing mature, we anticipate that applications of these methods to human microbiome samples will illuminate mobile element diversity within other complex microbial communities and enable further investigation into particular elements that may influence fitness under different environmental stresses.

## Conclusions

We demonstrate that read cloud assembly can capture strain-level genomic variants within metagenomic samples. We also show that different strain variants of microbial species fluctuate in their relative abundance over a relatively short time period in human microbiomes. These strain-level dynamics are associated with increased antibiotic resistance over the course of clinical treatment. Our work shows the potential of culture-free metagenomic sequencing approaches in investigating adaptive regulatory variations mediated by mobile elements and exploring strain dynamics within the gut microbiome.

## Supplementary information

**Additional file 1.** This file contains Figs. S1-S7, Tables S5, S6, S7, S10, and Supplementary results.

**Additional file 2.** Table S1. Species-level classification of the short read sequencing data for the four timepoints. The relative abundance of the species in the samples was determined after removing human and viral reads from the data. A subset of these data (the top 9 species in the samples) are shown in Fig. 1.

**Additional file 3.** Table S2. Species-level and genus-level classification of all the sequencing data for the four timepoints In this study. The relative abundance of the taxa in the samples was determined without removing human and viral reads from the data. Stacked bar graphs of these data are shown in Additional file 1: Fig. S1.

**Additional file 4.** Table S3. Assembly statistics, completeness metrics, tRNA and rRNA loci counts, total annotated genes, and coverage depths for all annotated species draft genomes in short-read and read cloud libraries from each time point. Results are shown for the largest bin of each species.

**Additional file 5. **Table S4. Assembly statistics, completeness metrics, tRNA and rRNA loci counts, total annotated genes, and coverage depths for bins created by merging all those annotated as *B. caccae* in short-read and read cloud libraries at each time point.

**Additional file 6. **Table S8. Coding sequences with RNA sequencing read counts and fold-change between time points in the neighboring 10 kb around the five IS614 integration loci in *B. caccae* estimated to have large-scale ancestral strain shifts. Gene annotations were obtained using Prokka. The target gene downstream of the putative promoter as well as the upstream gene are highlighted (green: downstream, red: upstream).

**Additional file 7.** Table S9. Total reads, sequencing coverage, assembled genome draft size and N50, and taxonomic annotation for all 53 isolates from stool samples of time points A, C, and D.

## Data Availability

The datasets supporting the conclusions of this article are available in the in the NCBI Sequence Read Archive under Bioproject accession PRJNA434731 [https://www.ncbi.nlm.nih.gov/bioproject/?term=PRJNA434731] [27]. The Athena assembler together with a demonstration dataset can be found at https://github.com/abishara/athena_meta [80]. The workflows used to produce and evaluate bins and generate the circos genome plot and composition barplot can be found at https://github.com/bhattlab/metagenomics_workflows [81].

## Abbreviations

DNA: Deoxyribonucleic acid
HCT: Hematopoietic cell transplantation
GI: Gastrointestinal
GVHD: Graft vs. host disease
SNV: Single-nucleotide variant
RNA: Ribonucleic acid
IS: Insertion sequence
MIC: Minimum inhibitory concentration
TYG: Tryptone yeast glucose
