# Supplementary material for: Strain-resolved microbiome sequencing reveals mobile elements that drive bacterial competition on a clinical timescale
Source: Genome Med. 2020 May 29;12:50. doi: 10.1186/s13073-020-00747-0 (PMC7260799; doi:10.1186/s13073-020-00747-0)
Supplement: Supplementary file 1 — Additional file 1. This file contains Figs. S1-S7, Tables S5, S6, S7, S10, and Supplementary results. [file 13073_2020_747_MOESM1_ESM.docx]

Additional file 1

[**Supplementary Figures and Tables**](#_q0xqpygmscvx) **2**

[**Supplementary Results**](#_67jr2dd7aif8) **15**

## Supplementary Figures and Tables

**
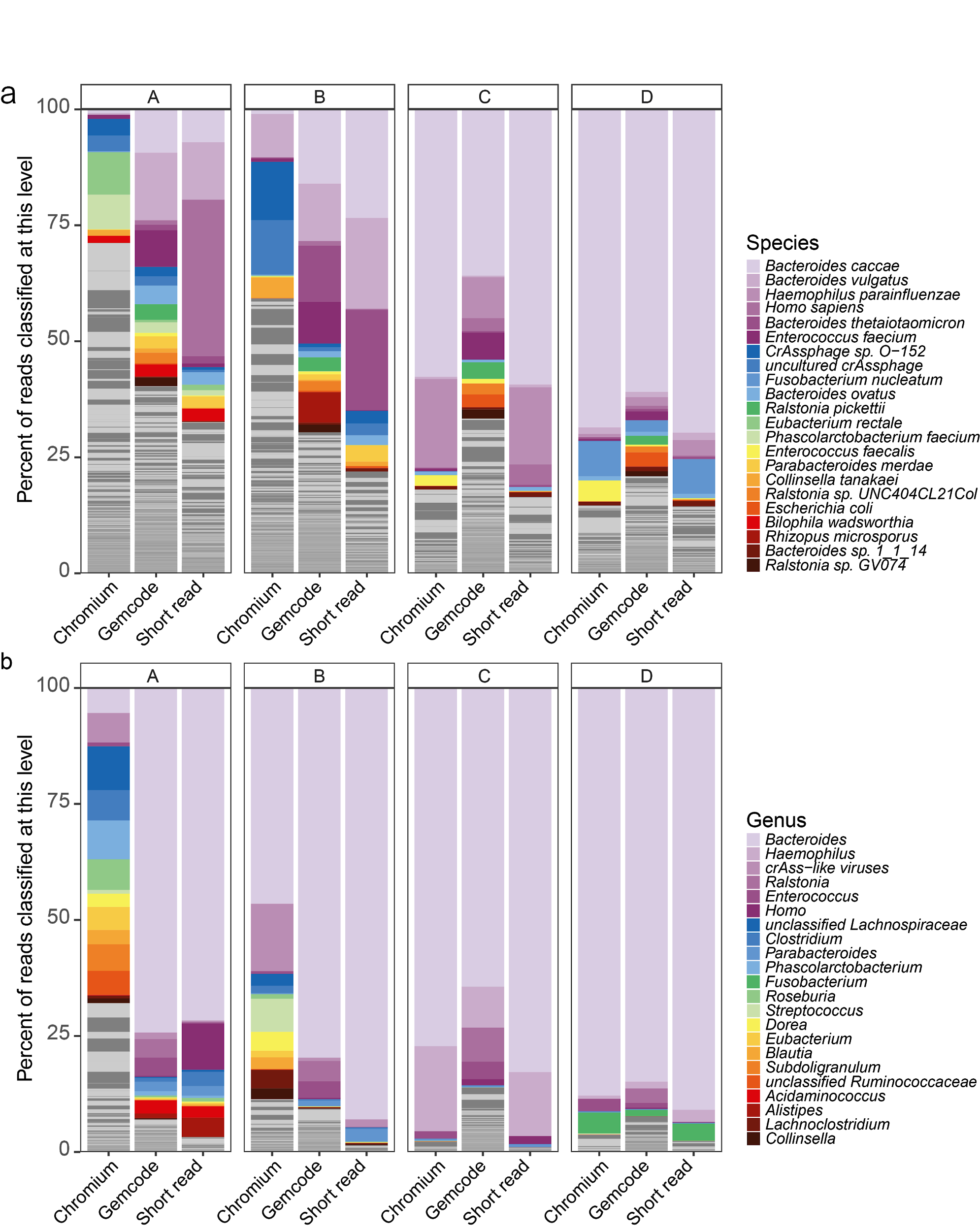
**

**Figure S1**

Species-level (a) and genus-level (b) taxonomic composition of the patient stool time series for Illumina Truseq short-read libraries, primary read cloud libraries prepared using the 10X Chromium platform, and previous read cloud libraries prepared using the now discontinued 10X Gemcode platform. For visual clarity, assignments for only the top 22 most abundant species and genera are identified. Reads receiving no classification, as well as reads classified at broader taxonomic levels than genus, are omitted for visual clarity. Human and viral genomes were included in the relative abundance calculations in these samples. The discrepancies in community composition across the methods is most likely due to differences in the DNA extraction protocols used. The short-read libraries and previous read cloud libraries, which were prepared with the 10X Genomics Gemcode platform, all used DNA extracted with mechanical lysis. These libraries displayed concordant species-level community composition across all samples. The primary read cloud libraries prepared with the more recent 10X Genomics Chromium platform were prepared from DNA extracted with enzymatic lysis (no mechanical lysis). Reads classified as *Ralstonia* were only observed in the 10X Gemcode libraries and are thus believed to be attributable to contamination in the Gemcode library. Relative abundance data for the species and genera used to generate the figures are available in Additional file 3: Table S2.


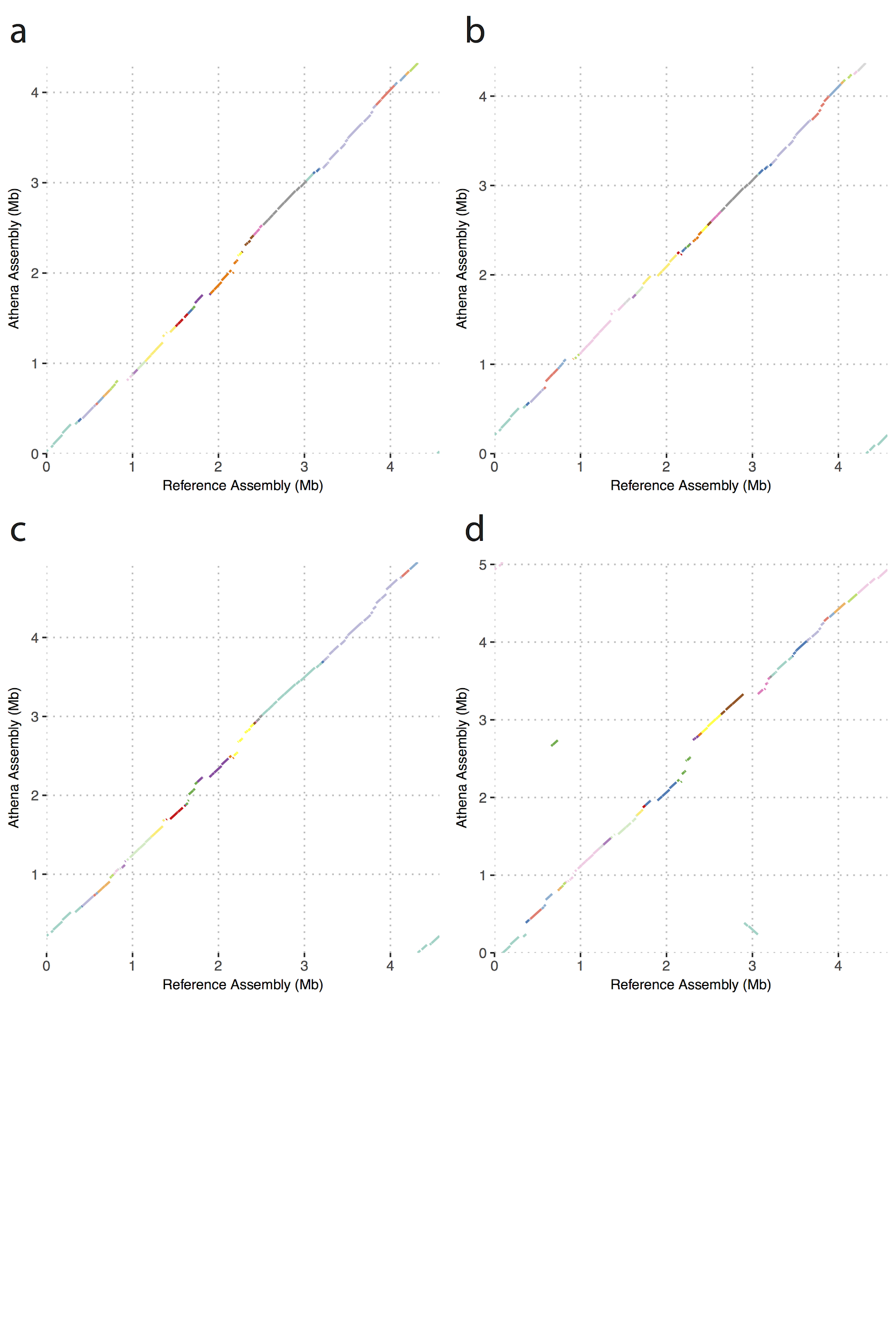


**Figure S2**

Dot-plot alignments between *B. caccae* read cloud drafts from the four time points (A, B, C, and D) against the available closed reference isolate genome (Genbank ID GCF_002222615.2).


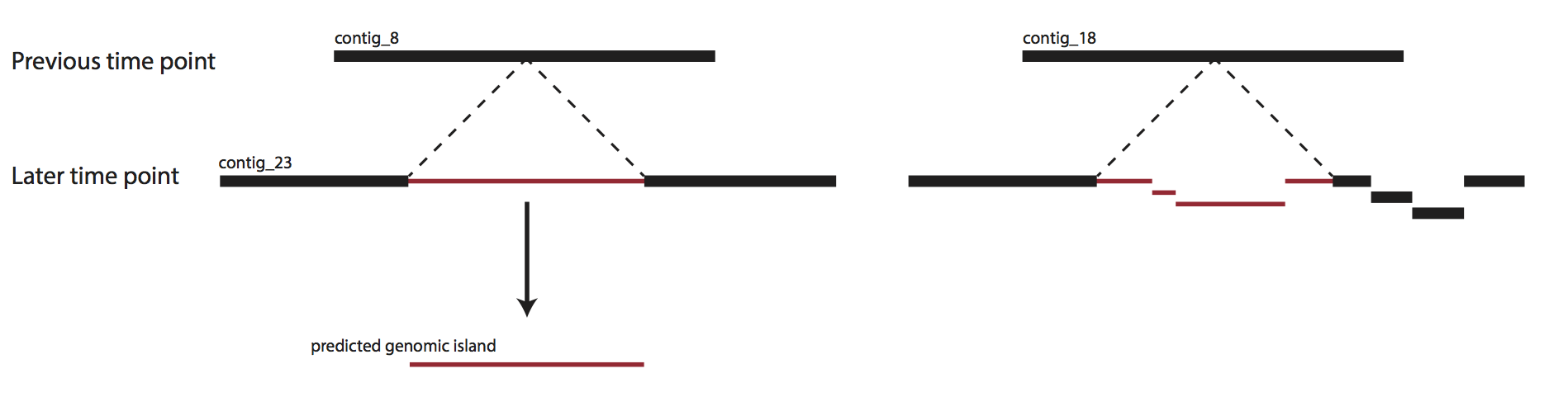


**Figure S3**

Alignments between single contigs before and after genomic island integration allow prediction of genomic island sequence (left). Genomic island sequences that are not fully assembled into a single contig within their genomic contexts in the later time point cannot be accurately predicted (right).


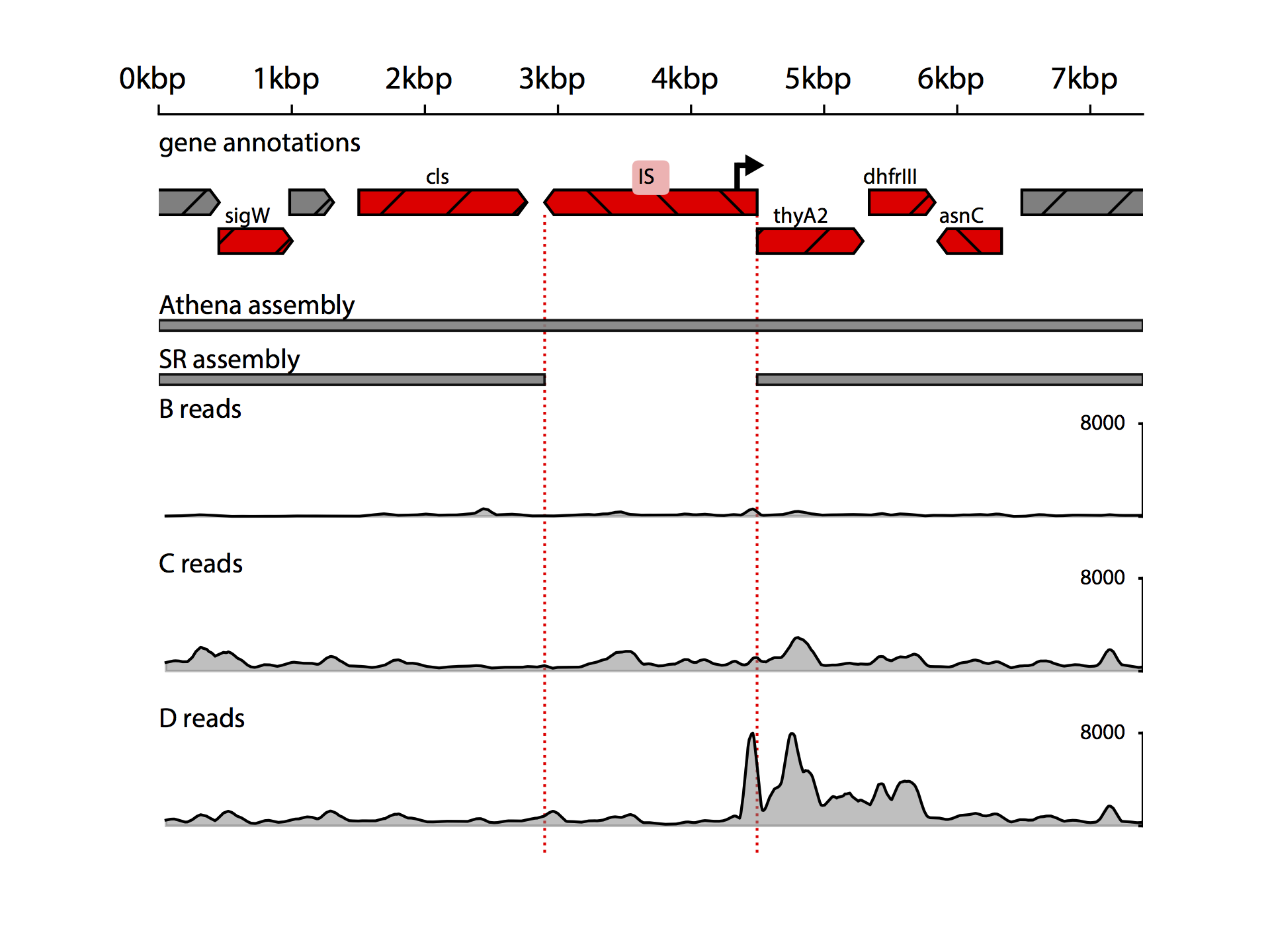


**Figure S4**

Metagenomic RNA sequencing supports IS-mediated transcription within *B. caccae.* Example of IS-mediated transcription for an additional gene, *thyA2*. Dominant strains in time point D harbor an introduced promoter and in the preceding time points B and C they do not. The transcriptional contribution of the IS is supported by increased RNA sequencing read depth in downstream genes relative to upstream genes, which coincides with an increase in the proportion of strains harboring the IS.


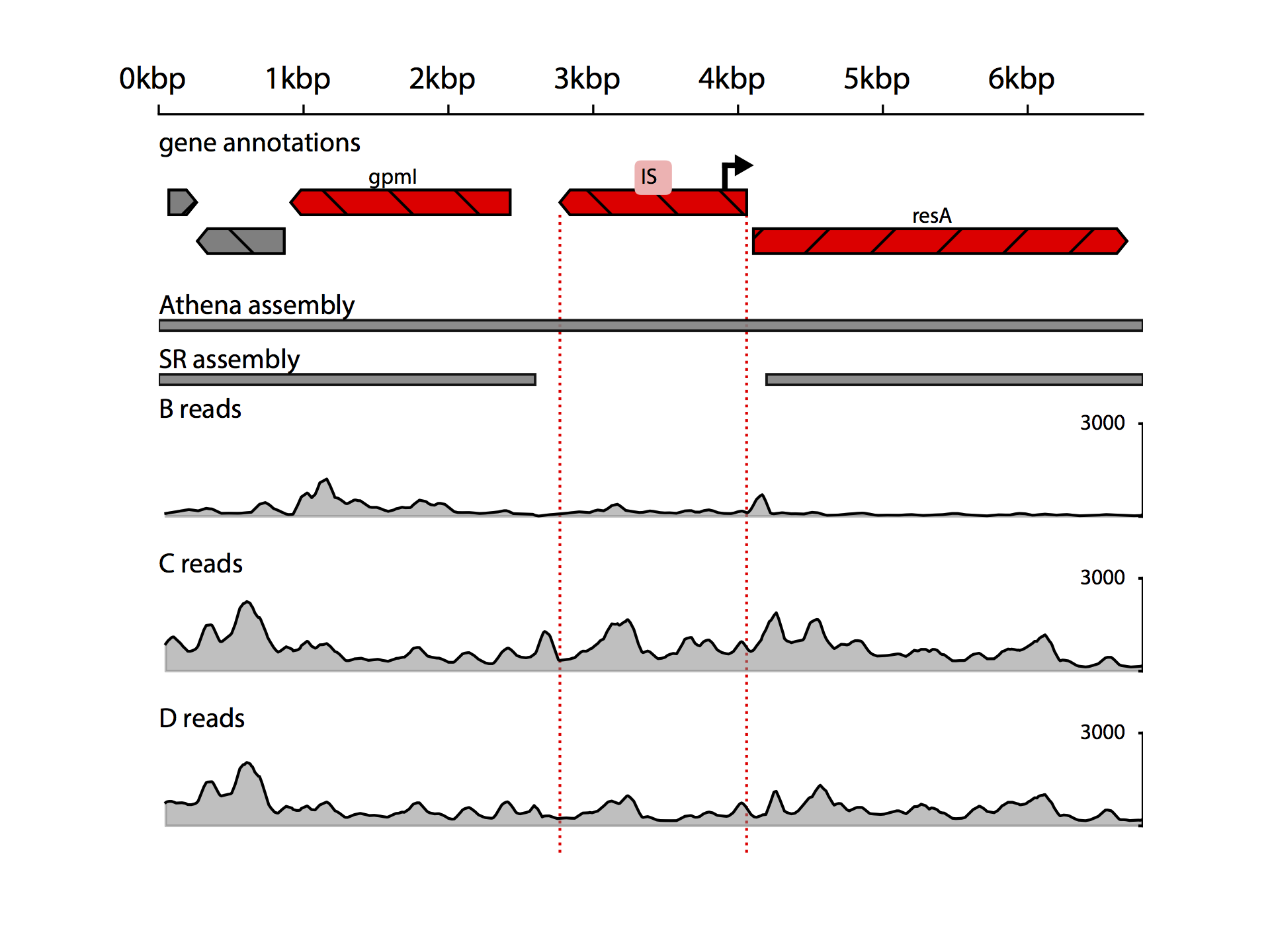


**Figure S5**

Metagenomic RNA sequencing supports IS-mediated transcription within *B. caccae.* Example of IS-mediated transcription for an additional gene, *resA*. Dominant strains in time point C harbor an introduced promoter and in the preceding time point B they do not. The transcriptional contribution of the IS is supported by increased RNA sequencing read depth in downstream genes relative to upstream genes, which coincides with an increase in the proportion of strains harboring the IS.


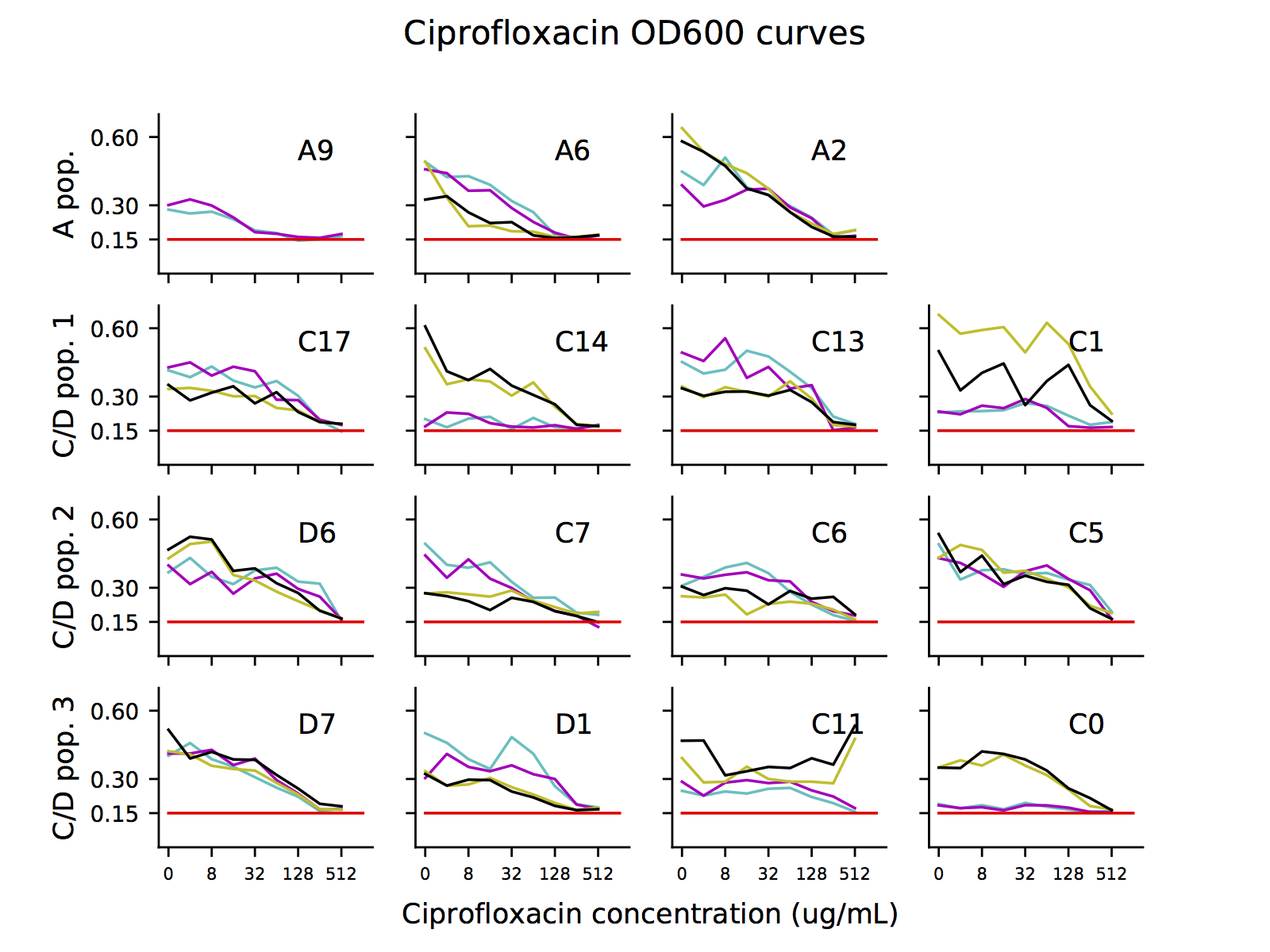


**Figure S6**

Raw OD600 readings of each tested *B. caccae* isolate strain against different concentrations of ciprofloxacin. Each strain was tested using two to four replicates. Strains are organized by their subpopulation assignment as determined by whole genome sequencing. Replicates showing minimal growth over a blank OD600 reading of sterile TYG media were excluded from the MIC analysis. Although higher concentrations of the drug were tested, OD600 readings above 512 µg/mL were excluded as ciprofloxacin was observed to precipitate at higher concentrations after the 48 hour incubation period of the assay.


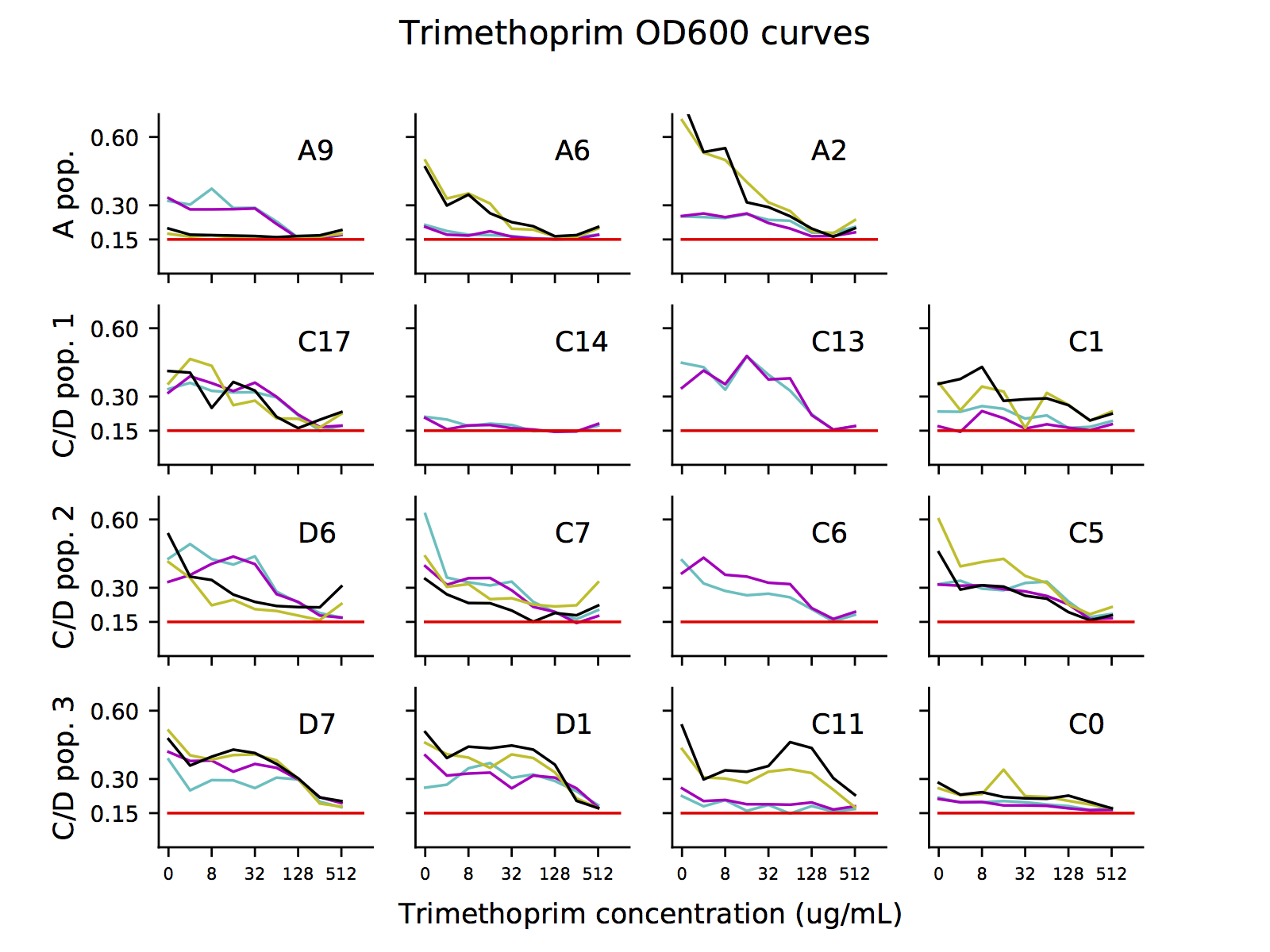


**Figure S7**

Raw OD600 readings of each tested *B. caccae* isolate strain against different concentrations of trimethoprim. Each strain was tested using two to four replicates. Strains are organized by their subpopulation assignment as determined by whole genome sequencing. Replicates showing minimal growth over a blank OD600 reading of sterile TYG media were excluded from the MIC analysis. OD600 readings above 512 µg/mL were excluded as trimethoprim was observed to precipitate at higher concentrations after the 48 hour incubation period of the assay.

| reference Genbank ID | draft | reference length | draft length | median sequence identity (%) | unaligned reference bases | unaligned draft bases |
| --- | --- | --- | --- | --- | --- | --- |
| NZ_JH724079.1 | Read cloud | 5493117 | 5476141 | 99.175 | 1078924 | 1076271 |
| NZ_CP022412.2 | Read cloud | 4570803 | 5476141 | 99.474 | 513456 | 1431898 |
| NZ_AAVM02000021.1 | Read cloud | 4564814 | 5476141 | 99.479 | 515847 | 1437337 |
| NZ_PUEQ01000001.1 | Read cloud | 4577788 | 5476141 | 99.479 | 528503 | 1434141 |
| NZ_CZBL01000001.1 | Read cloud | 5291863 | 5476141 | 99.484 | 910158 | 1099942 |
| NZ_CZAI01000001.1 | Read cloud | 5337582 | 5476141 | 99.633 | 733275 | 899825 |
| combined | Read cloud |  | 5476141 |  |  | 638951 |
| NZ_JH724079.1 | Short read | 5493117 | 4713495 | 99.263 | 1348485 | 602650 |
| NZ_CP022412.2 | Short read | 4570803 | 4713495 | 99.486 | 654106 | 796989 |
| NZ_AAVM02000021.1 | Short read | 4564814 | 4713495 | 99.485 | 658494 | 797371 |
| NZ_PUEQ01000001.1 | Short read | 4577788 | 4713495 | 99.486 | 671140 | 796988 |
| NZ_CZBL01000001.1 | Short read | 5291863 | 4713495 | 99.683 | 1181934 | 648442 |
| NZ_CZAI01000001.1 | Short read | 5337582 | 4713495 | 99.692 | 1047949 | 464972 |
| combined | Short read |  | 4713495 |  |  | 317696 |

**Table S5**

Median sequence identity and total number of unaligned bases between *B. caccae* drafts from time point C drafts and six available reference isolate genomes.

| Time point | Read cloud draft location | Size | Other organisms |
| --- | --- | --- | --- |
| C | contig_3:715785-732470 | 16685 | B. vulgatus and B. uniformis |
| C | contig_3:849029-906491 | 57462 | B. vulgatus |
| C | scaffold_20:157993-228816 | 70823 |  |
| D | contig_67:129068-179225 | 50157 |  |
| D | contig_67:60290-102957 | 42667 |  |

**Table S6**

The locations of large-scale genomic islands within the assembled read cloud drafts detected by pairwise sequence alignments of *B. caccae* drafts from successive time points. Two of these islands were also found to be present within the draft genomes of other organisms present in the samples.

| Source time point draft | Read cloud draft IS612 location | A | B | C | D | downstream gene (where applicable) |
| --- | --- | --- | --- | --- | --- | --- |
| a | contig_133:369670-371266 | 0.452 | 0.513 | 0.646 | 0.760 | resA |
| a | contig_3:281420-283016 | 0.000 | 0.000 | 0.000 | 0.000 |  |
| a | contig_34:3881-5477 | 0.000 | 0.000 | 0.000 | 0.000 | per1 |
| a | contig_34:30545-32141 | 0.688 | 0.573 | 0.709 | 0.685 |  |
| a | contig_366:11196-12792 | 0.220 | 0.585 | 0.756 | 0.669 |  |
| a | contig_452:35256-36852 | 0.051 | 0.015 | 0.022 | 0.672 |  |
| a | contig_70:225046-226642 | 0.094 | 0.016 | 0.044 | 0.666 |  |
| a | contig_70:315579-317175 | 0.033 | 0.008 | 0.014 | 0.288 |  |
| a | contig_87:7379-8975 | 0.000 | 0.000 | 0.000 | 0.000 |  |
| a | contig_87:36950-38546 | 0.021 | 0.025 | 0.027 | 0.554 |  |
| b | contig_171:85806-87402 | 0.000 | 0.000 | 0.000 | 0.000 |  |
| b | contig_388:58644-60240 | 0.034 | 0.013 | 0.022 | 0.556 |  |
| c | contig_2:345613-347209 | 0.044 | 0.025 | 0.018 | 0.561 |  |
| c | contig_21:136948-138544 | 0.583 | 0.854 | 0.136 | 0.583 |  |
| c | contig_3:133306-134902 | 0.515 | 0.594 | 0.162 | 0.630 |  |
| c | contig_3:713286-714882 | 0.305 | 0.834 | 0.164 | 0.743 |  |
| d | contig_18:332510-334106 | 0.623 | 0.826 | 0.674 | 0.149 | thyA2 |
| d | contig_4:129400-130996 | 0.491 | 0.723 | 0.000 | 0.000 | norM |

**Table S7**

Estimated fractional abundances of ancestral strains at each assembled *B. caccae* insertion site. Reads originating from strains without a given IS instance (ancestral strains) are recognized by having gapped alignments across the assembled insertion sequence. Ancestral strain fraction is expressed as the number of observed gapped alignments over the median sequence coverage within the neighboring 10kb of sequence. Also noted are adjacent genes mentioned in the main text that are downstream of the putative outward-facing promoter carried on the insertion sequence.

| Library | Time point | Total Reads | Total Bases | Total Bases (Gb) | Total Reads w/QC | Total Bases w/QC | Total Bases w/QC (Gb) |
| --- | --- | --- | --- | --- | --- | --- | --- |
| Short read | A | 366,125,362 | 34,549,490,342 | 34.55 | 335,984,623 | 31,582,554,562 | 31.58 |
| Short read | B | 57,939,592 | 5,909,838,384 | 5.91 | 53,327,906 | 5,187,200,671 | 5.19 |
| Short read | C | 66,691,350 | 6,802,517,700 | 6.80 | 61,961,698 | 6,035,573,394 | 6.04 |
| Short read | D | 27,814,734 | 2,837,102,868 | 2.84 | 25,920,034 | 2,521,145,129 | 2.52 |
| Chromium | A | 147,103,476 | 20,594,486,640 | 20.59 | 144,405,692 | 19,855,169,050 | 19.86 |
| Chromium | B | 140,586,910 | 19,682,167,400 | 19.68 | 138,156,773 | 19,033,140,760 | 19.03 |
| Chromium | C | 135,132,378 | 18,918,532,920 | 18.92 | 132,255,256 | 18,201,215,198 | 18.20 |
| Chromium | D | 152,224,794 | 21,311,471,160 | 21.31 | 149,329,401 | 20,575,219,967 | 20.58 |
| Gemcode | A | 49,121,872 | 7,073,549,568 | 7.07 | 23,607,499 | 997,034,214 | 1.00 |
| Gemcode | B | 39,171,402 | 5,640,681,888 | 5.64 | 17,394,123 | 749,959,878 | 0.75 |
| Gemcode | C | 29,894,142 | 4,304,756,448 | 4.30 | 11,665,434 | 564,141,282 | 0.56 |
| Gemcode | D | 40,573,138 | 5,842,531,872 | 5.84 | 19,771,511 | 944,012,183 | 0.94 |
| RNA | A | - | - | - | - | - | - |
| RNA | B | 118,775,928 | 12,115,144,656 | 12.12 | - | - | - |
| RNA | C | 83,575,948 | 8,524,746,696 | 8.52 | - | - | - |
| RNA | D | 79,094,650 | 8,067,654,300 | 8.07 | - | - | - |

**Table S10**

Total reads and sequencing coverage for all metagenomic sequencing libraries before and after quality control.

**Additional file 2-Table S1 (separately attached)**

Species-level classification of the short read sequencing data for the four timepoints. The relative abundance of the species in the samples was determined after removing human and viral reads from the data. A subset of these data (the top 9 species in the samples) are shown in Figure 1.

**Additional file 3-Table S2 (separately attached)**

Species-level and genus-level classification of all the sequencing data for the four timepoints In this study. The relative abundance of the taxa in the samples was determined without removing human and viral reads from the data. Stacked bar graphs of these data are shown in Additional file 1: Fig. S1.

**Additional file 4-Table S3 (separately attached)**

Assembly statistics, completeness metrics, tRNA and rRNA loci counts, total annotated genes, and coverage depths for all annotated species draft genomes in short-read and read cloud libraries from each time point. Results are shown for the largest bin of each species.

**Additional file 5-Table S4 (separately attached)**

Assembly statistics, completeness metrics, tRNA and rRNA loci counts, total annotated genes, and coverage depths for bins created by merging all those annotated as *B. caccae* in short-read and read cloud libraries at each time point.

**Additional file 6-Table S8 (separately attached)**

Coding sequences with RNA sequencing read counts and fold-change between time points in the neighboring 10kb around the five IS614 integration loci in *B. caccae* estimated to have large-scale ancestral strain shifts. Gene annotations were obtained using Prokka. The target gene downstream of the putative promoter as well as the upstream gene are highlighted (green: downstream, red: upstream).

**Additional file 7-Table S9 (separately attached)**

Total reads, sequencing coverage, assembled genome draft size and N50, and taxonomic annotation for all 53 isolates from stool samples of time points A, C, and D.

## Supplementary Results

IS614 adjacent to *per1*

The rise in expression of *per1* coincided with a one-week course of cefepime followed immediately by a two week course of meropenem between timepoints B and C. *Per1* continues to exhibit high expression in time point D, 19 days after withdrawal of meropenem, despite the absence of any further beta-lactam antibiotic administration. Our drafts located an IS adjacent to this gene oriented correctly for IS-mediated transcription to occur. While estimating relative abundance of this insertion, we were unable to detect reads from the ancestral strain, and determined this insertion to be fixed within the population over the course of treatment. This particular instance of *per1* and neighboring sequence was only found using our read cloud approach and was not present in any of the six available reference isolate *B. caccae* genomes or in any of the short-read assembled *B. caccae* drafts.

Gram-positive enrichment in DNA extractions with enzymatic lysis

The short-read libraries and previous read cloud libraries, which were prepared with the now discontinued 10X Genomics Gemcode platform, all utilized DNA extracted with mechanical lysis. These libraries displayed concordant species-level community composition across all samples (Supplementary Figure 1). Our primary read cloud libraries prepared with the current 10X Genomics Chromium platform, were prepared from DNA extracted with enzymatic lysis and show a greater representation of gram positive bacteria. This suggests that the differing extraction protocols used for the primary read cloud and short-read libraries, not the library preparation method, are the main source of discrepancies in community composition.
